# Une dermatose auto provoquée de l’épaule

**DOI:** 10.11604/pamj.2018.30.149.14708

**Published:** 2018-06-20

**Authors:** Mohamed El Amraoui, Badredine Hassam

**Affiliations:** 1 Service de Dermatologie, Hôpital Avicenne, CHU Ibn Sina, Rabat, Maroc

**Keywords:** Bulles, épaule, brulure, dépression, Bubbles, shoulder, burns, depression

## Abstract

Self-induced dermatoses are more and more frequent in adolescent substance abusers with adaptation problems. We here report a case of self-induced bullous lesions on the shoulder in an adolescent with major depression and suicidal ideations. The study involved a 22-year old man, who was a chronic smoker, an occasional user of cannabis and alchool with no particular past medical history, with divorced parents, in conflict with his father. He presented with bullous eruption on the left shoulder made of bubbles, post-bullous skin erosions and scabs on a healthy skin. The monomorphic appearance of lesions, their accessibility (on the left shoulder in a right-handed patient), patient’s history and his psychiatric problems have immediately suggested the investigation of self-induced dermatosis or factitious disorder. The patient admitted that he had taken psychotropic drugs and that the self-induced lesions had been caused by cigarette burns. This first psichologic evaluation also showed that the patient had suicidal ideations and a self-and hetero-aggressive behavior. The patient was treated with emollients and healing creams and referred to the Department of Psychiatry for complementary therapies.

## Image en médecine

Les dermatoses auto provoquées sont de plus en plus fréquentes chez les adolescents toxicomanes avec des difficultés adaptatives. Nous présentons un cas de lésions bulleuses de l'épaule auto provoquées chez un adolescent ayant révélé un état dépressif majeur avec des idées suicidaires. Jeune homme âgé de 22 ans, sans antécédents pathologiques notables, ayant un tabagisme chronique, un cannabisme et un alcoolisme occasionnels, ses parents sont divorcés avec des conflits avec son père. A consulté pour une éruption bulleuse de l’épaule gauche faite de bulles, d’érosions post bulleuses et des croutes, siégeant sur une peau saine. L’aspect monomorphe des lésions, l’accessibilité (la localisation sur l’épaule gauche chez un droitier), les antécédents du patient et ses difficultés psychiatriques ont rapidement orienté l’enquête vers la possibilité d’une dermatose auto-provoquée ou une pathomimie. Le patient avouait la prise de psychotropes et que les lésions ont été auto provoquées par des brulures de cigarette. Ce premier abord psychologique a révélé également la présence d’idées suicidaires et un comportement d’auto et d’hétéro agressivité. Le patient a été mis sous émollients et crèmes cicatrisantes et adressé en consultation de psychiatrie de l’hôpital pour complément de prise en charge.

**Figure 1:**
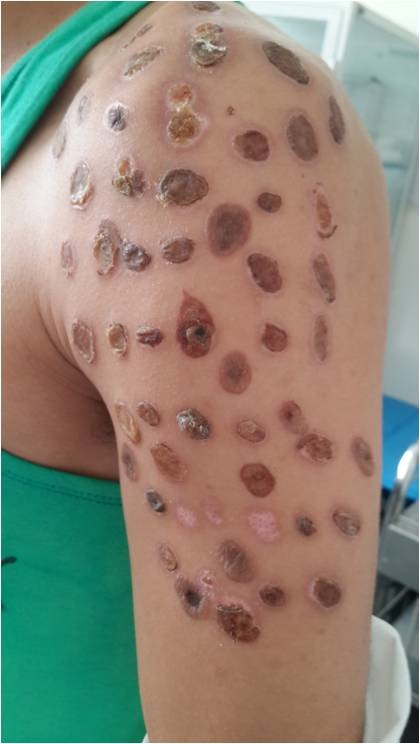
Brûlures auto provoquées chez un adolescent toxicomane avec des difficultés psychiatriques

